# How adverse and benevolent childhood experiences influence depression and suicidal ideation in Chinese undergraduates: a latent class analysis

**DOI:** 10.1265/ehpm.22-00242

**Published:** 2023-02-22

**Authors:** Jie Tang, Jingjing Wang, Yifei Pei, Shiferaw Blen Dereje, Qian Chen, Na Yan, Yunjiao Luo, Yuhao Wang, Wei Wang

**Affiliations:** 1 School of Public Health, Xuzhou Medical University, 209 Tong Shan Road, Xuzhou, 221004, Jiangsu, China; 2 Key Laboratory of Human Genetics and Environmental Medicine, Xuzhou Medical University, Xuzhou, China; 3 Engineering Research Innovation Center of Biological Data Mining and Healthcare Transformation, Xuzhou Medical University, Xuzhou, Jiangsu, 221004, China

**Keywords:** Benevolent childhood experiences, Adverse childhood experiences, Psychological distress, Undergraduates

## Abstract

**Background:**

There has been minimal research on the role of benevolent childhood experiences (BCEs) and how such events may offer protection from the insidious effects of adverse childhood experiences (ACEs) or later in life.

**Objectives:**

This research aims to learn how BCEs and ACEs interact to affect adolescents’ psychological distress.

**Methods:**

Cross-sectional survey was conducted in three cities (Xuzhou, Nanjing, and Wuhan) in China from March 2021 to May 2021. Latent class analysis (LCA) was used to classify the patterns of ACEs and BCEs. We adopted hierarchical multivariable regression to examine the influences of ACEs and BCEs on depression and suicidal ideation.

**Results:**

To explore the relationship between childhood experience and suicidal ideation and depression, LCA revealed three patterns of ACEs: (1) emotional abuse (10.57%); (2) high ACEs (0.55%); and (3) low ACEs classes (88.88%). Adolescents with emotional abuse (depression: OR = 3.82, 95%CI = 2.80–5.22, *P* < 0.001; suicidal ideation: OR = 5.766, 95%CI = 3.97–8.38, *P* < 0.001) and high ACEs class (suicidal ideation: OR = 5.93, 95%CI = 1.19–29.66, *P* < 0.05) had an increased risk of psychological distress (reference: low ACEs). LCA revealed four patterns of BCEs: (1) relationship support (14.54%); (2) low BCEs (4.85%); (3) high BCEs (55.34%); and (4) high quality of life classes (25.28%). Adolescents with a high quality of life (depression: OR = 0.09, 95%CI = 0.05–0.16, *P* < 0.001; suicidal ideation: OR = 0.22, 95%CI = 0.12–0.40, *P* < 0.001) and high BCEs (depression: OR = 0.05, 95%CI = 0.03–0.09, *P* < 0.001; suicidal ideation: OR = 0.15, 95%CI = 0.09–0.26, *P* < 0.001) protected the mental health of adolescents (reference: low BCEs).

**Conclusions:**

High ACEs and emotional abuse classes were significantly associated with poorer mental health symptoms, including suicidal ideation and depression. In contrast, high BCEs and high quality of life classes were associated with better mental health. These findings point out that it is more necessary to identify and support victims of ACEs, and it is urgent to increase BCEs in early childhood.

## Introduction

Depression and suicidal ideation are serious public health problems worldwide [1]. Existing studies indicated that the prevalence of depression among adolescents ranges from 10% to 85%, with a weighted average prevalence of 30.60% [2]. In addition, data from the World Health Organization estimates that more than 0.8 million people die from suicidal ideation globally each year [3]. Many studies have found a link between adolescent psychological distress and factors such as violence, unintentional injury, poor sleep quality, and bullying [4, 5]. However, in addition to these factors, it is worth noting that psychological distress can also be caused by childhood experiences [6]. An increasing number of studies suggest that childhood experiences are essential for people’s health throughout their lives [7, 8], including adverse childhood experiences (ACEs) and benevolent childhood experiences (BCEs) [9, 10].

The relationship between ACEs (e.g., child abuse [7], parental divorce [11], and parental mental illness [12]) and a wide range of poorer health outcomes has been studied extensively [13]. According to previous studies, 39% of adolescents have at least one type of ACE, and Chinese undergraduates with a history of ACEs are more likely to report symptoms of psychological distress and ultimately higher premature mortality than those without a history of ACEs [14, 15]. Given the harmful effects of ACEs on health, recent studies have begun to identify protective factors that can help adolescents overcome the negative impacts of ACEs on mental health outcomes [16, 17]. Indeed, positive self-awareness and relationship with caregivers, peers, and teachers are known as BCEs [18]. Contrary to ACEs, BCEs represent positive early experiences before 18 years old [19], including having at least one safe caregiver, having one or more close friends, and having a predictable family routine [19, 20]. To date, most studies have reported higher BCEs with lower symptoms of stress, depression, and PTSD [18, 19, 21]. It is necessary to understand how BCEs and ACEs affect the psychological distress of adolescents as a whole [22].

Numerous studies relied on cumulative risk scores or personal adversity as measured by retrospective self-reports [17, 23, 24]. These methods, however, have significant limitations. On the contrary, latent class analysis (LCA) is a data-driven approach designed to identify different groups or categories of individuals with similar reported patterns of adversity [13], which can provide a replaceable system to operate ACEs and BCEs. There have been some studies that started to use LCA to discover distinctive patterns of ACEs [25]. However, few studies used LCA to identify distinct patterns of BCEs and to investigate the relationship between the pattern of ACEs and BCEs and psychological distress simultaneously.

This research aims to classify BCEs and ACEs using LCA and investigate the relationship between childhood experience patterns and depression and suicidal ideation. Our study provides a scientific foundation and theoretical rationale for psychological distress among adolescents to enhance teenagers’ mental health.

## Methods

### Procedures and participants

The cross-sectional survey was conducted in three cities (Xuzhou, Nanjing, and Wuhan) in China from March 2021 to May 2021. We used a cluster random sampling method to get a representative sample from 25 universities in three cities (i.e., Xuzhou, Nanjing, and Wuhan) in three provinces by using an online questionnaire. We determined the number of universities in each city according to the size of the city, and then we randomly selected universities in each city.

Participants completed an anonymous electronic questionnaire via WeChat. There were no incentives, and each participant was informed of their right to withdraw first from the survey. We excluded unreliable or unqualified questionnaires (logic errors and answer time less than 600 seconds) for quality control. For most participants, it took about 30 minutes to perform the questionnaires. A total of 2022 students participated in this survey; after excluding the sample with incomplete data for key variables (n = 206), 1816 students were successfully included in the final analysis, with an excellent response rate (89.8%). And all participants provided written informed consent.

The Ethics Committee of Xuzhou Medical University granted ethical approval for this study. The participants provided their written informed consent to participate in this study.

### Measures

#### Demographic characteristics

Basic characteristics including age, gender (male or female), grades (freshman, sophomore, junior, senior), living expenses (yuan) (≤1000, 1001–2000, 2001–3000, >3000), only-child (yes or no), residence (urban or rural) and sexual orientation (heterosexual, homosexual, bisexuality, others).

#### Benevolent childhood experiences

The BCEs were measured by the Chinese BCEs Scale, which verified the validity and reliability of previous studies [18, 19]. The scale contains ten items, self-report the positive experiences from birth to 18 years old. Standard questionnaires such as (1) perceived internal and external security (e.g., having beliefs that gave comfort); (2) positive and predictable quality of life (e.g., having opportunities to have a good time); and (3) relationship support (e.g., having at least one good friend). Each “yes” response indicated that the participant had the BCE and counted as “1” point, while a “No” response resulted in “0” points. The Cronbach’s Alpha in our sample was 0.729. The total score of BCEs consists of 10 items (ranging from 0 to 10). The higher score reflects a more positive childhood experience.

#### Adverse childhood experiences

ACEs occurring before 18 years old were assessed using the Kaiser-CDC ACEs Scale [26, 27]. The scale includes three subscales and ten items, including abuse (e.g., emotional abuse), neglect (e.g., physical neglect), and family dysfunction (e.g., household members who are incarcerated). Each question contains two answers: “Yes” and “No.” Each “Yes” response received a “1,” while each “No” response received a “0,” and the Cronbach’s Alpha in our sample was 0.729. The sum of ten items produced the total score (range 0–10). The higher the score, the greater the risk of adverse events.

#### Depression

A 10-item questionnaire (CESD-10), a shortened version of the CESD-20, was used to assess depression in the previous week. The scale was rated on a Linkert scale ranging from 0 (rarely or never, <1 day) to 3 (all the time, 5 to 7 days). Items 5 and 8 are scored in inverse order. The total score of 10 items is calculated to assess the depressed mood, and the higher score represents higher depression [28, 29]. The Cronbach’s α of the scale was 0.869, which is acceptable in our study. A cut-off point of 10 has been adopted in this study to define elevated depression [30].

#### Suicidal ideation

Self-reported SI was based on responding to the following question: “Did you seriously consider suicidal ideation during the past month?” Options were dichotomous: yes or no.

### Statistical analysis

Descriptive analysis and Logistic regression performed by SPSS 25.0 was used to describe the basic characteristics. LCA based on Mplus 8.0 was performed to generate the latent classes of ACEs and BCEs. We selected the best-classified model through Akaike’s Information Criterion (AIC), Bayesian Information Criterion (BIC), Adjusted Bayesian Information Criterion (ABIC), and Entropy. Smaller AIC, BIC, and ABIC values, and higher entropy indicated better model fit [31]. Chi-square tests examined the relationships between demographic factors and suicidal ideation and depression. Previous studies have confirmed that gender, living expenses, only-child, and residence were associated with adolescent mental health, so these factors were included in the multivariable logistic regression analysis to examine the relationship between childhood experiences and suicidal ideation and depression [32–35]. *P* < 0.05 was considered statistically significant, and a two-tailed test was used.

## Results

### Basic characteristics of the participants

The cross-sectional study was conducted among 1816 university students, as shown in Table 1, the average age of them was 20.08 years (SD = 1.17). Most students (79.19%) had living expenses between 1001–2000 yuan, and most were heterosexual (89.48%). Approximately half of the sample were only children. Table 1 also shows the relationship between depression and suicidal ideation with different socio-demographic characteristics. In univariate analysis, only sexual orientation was significantly related to depression (p = 0.02) and suicidal ideation (*p* < 0.01).

**Table 1:** Basic characteristics of the participants (n = 1816)

**Variables**	**Overall** **(n = 1816)**	**Depression**		**χ^2^/t**	***p***	**Suicidal ideation**		**χ^2^/t**	***p***
	
		**Yes(%)**	**No(%)**			**Yes(%)**	**No(%)**		
**Age(year)**									
20.08 ± 1.17		20.09 ± 1.14	20.07 ± 1.18	0.29	0.77	20.05 ± 1.17	20.08 ± 1.17	−0.28	0.78
**Gender**									
Male	554(30.51)	188(33.90)	366(66.10)	0.33	0.57	42(7.60)	512(92.40)	2.34	0.13
Female	1262(69.49)	411(22.60)	851(67.40)			124(9.80)	1138(90.20)		
**Grades**									
Freshman	278(15.31)	80(28.80)	198(72.10)	6.02	0.11	32(11.50)	246(88.50)	3.06	0.38
Sophomore year	829(45.65)	296(35.70)	533(64.30)			75(9.00)	754(91.00)		
Junior year	666(36.67)	211(31.70)	455(68.30)			54(8.10)	612(91.90)		
Senior year	43(2.37)	12(27.90)	31(72.10)			5(11.60)	38(88.40)		
**Living expenses**									
a1000	92(5.07)	33(35.90)	59(64.10)	0.97	0.82	12(13.00)	80(87.00)	3.94	0.27
1001–2000	1438(79.19)	468(32.50)	970(67.50)			135(9.40)	1303(90.60)		
2001–3000	226(12.44)	79(35.00)	147(65.00)			15(6.60)	211(93.40)		
a3000	60(3.30)	19(31.70)	41(3.468.30)			4(6.70)	56(93.30)		
**Only-child**									
Yes	906(49.89)	287(37.10)	619(68.30)	1.40	0.24	81(8.90)	825(91.1)	0.09	0.77
No	910(50.11)	312(34.30)	598(65.70)			85(9.30)	825(90.70)		
**Residence**									
Urban	1027(56.55)	345(33.60)	682(66.40)	0.40	0.53	104(10.10)	923(89.90)	2.77	0.10
Rural	789(43.45)	254(32.20)	535(67.80)			62(7.90)	727(92.10)		
**Sexual orientation**									
Heterosexuality	1625(89.48)	517(31.80)	1108(68.20)	9.89	0.02	126(7.80)	1499(92.20)	37.60	<0.01
Homosexuality	40(2.20)	17(42.50)	23(57.50)			8(20.00)	32(80.00)		
Bisexuality	108(5.95)	45(41.70)	63(58.30)			25(23.10)	83(76.90)		
Others	43(2.37)	20(46.50)	23(53.50)			7(16.30)	36(83.70)		

### Latent class analysis

Model selection statistics with one through five latent classes are summarized in Table 2. The AIC decreased as the number of classes increased. In the BCEs group, the three-class model had the lowest BIC, and the four-class model had the lowest ABIC. Although the five-class model had the highest entropy, the Lo-Mendell-Rubin likelihood ratio test (LMRT) had a non-significant *p*-value. Thus, we chose the four-class model. Similarly, in the ACEs group, the three-class model had the lowest BIC, the highest entropy, and relatively AIC and ABIC. As a result, the three-class model was chosen based on model fit and interpretability.

**Table 2:** LCA fit indices of childhood experiences among Chinese undergraduate

**Childhood experience Group**	**Number of Classes**	**AIC**	**BIC**	**ABIC**	**Entropy**	**BLRT**	**LMRT**	**Probability**
BCEs	1	13074.06	13129.104	13097.335	-	-	-	-
	2	11573.739	11689.331	11622.615	0.77	0	0	0.77808/0.22192
	3	11352.02	11528.161	11426.498	0.722	0	0	0.35077/0.59196/0.05727
	4	11298.922	11535.611	11399.002	0.743	0	0.0015	0.14537/0.04846/0.55341/0.25275
	5	11281.616	11578.853	11407.298	0.757	0	0.0739	0.52588/0.15914/0.25000/0.04075/0.02423

ACEs	1	6902.561	6957.605	6925.835	-	-	-	-
	2	5909.201	6024.793	5958.077	0.903	0	0	0.10573/0.89427
	3	5713.249	5889.39	5787.727	0.915	0	0.0003	0.10573/0.00551/0.88877
	4	5671.307	5907.996	5771.387	0.914	0	0.0256	0.13216/0.02203/0.84086/0.00496
	5	5653.426	5950.663	5779.108	0.913	0	0.0737	0.00496/0.01046/0.02203/0.13271/0.82985

Note: AIC = Akaike information criterion; BIC = Sample-size adjusted Bayesian Information Criterion; ABIC = sample-size adjusted Bayesian information criterion; BLRT = bootstrap likelihood ratio test; LMR = Adjusted Lo-Mendell-Rubin likelihood ratio test.

**Fig. 1:**
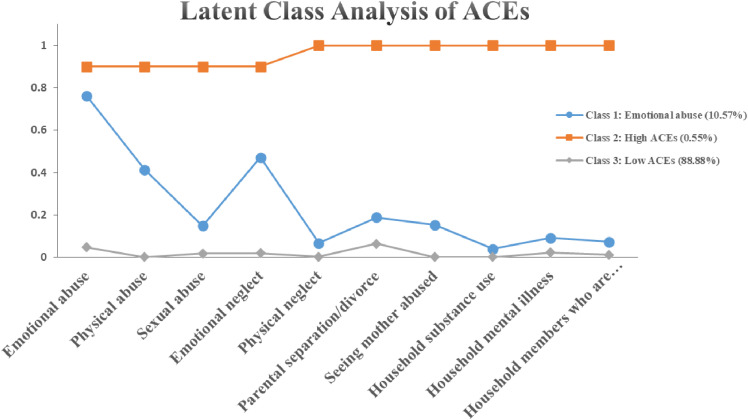
Probabilities of ACEs items by childhood experiences pattern derived by LCA Note: ACEs = adverse childhood experience; LCA = latent class analysis

**Fig. 2:**
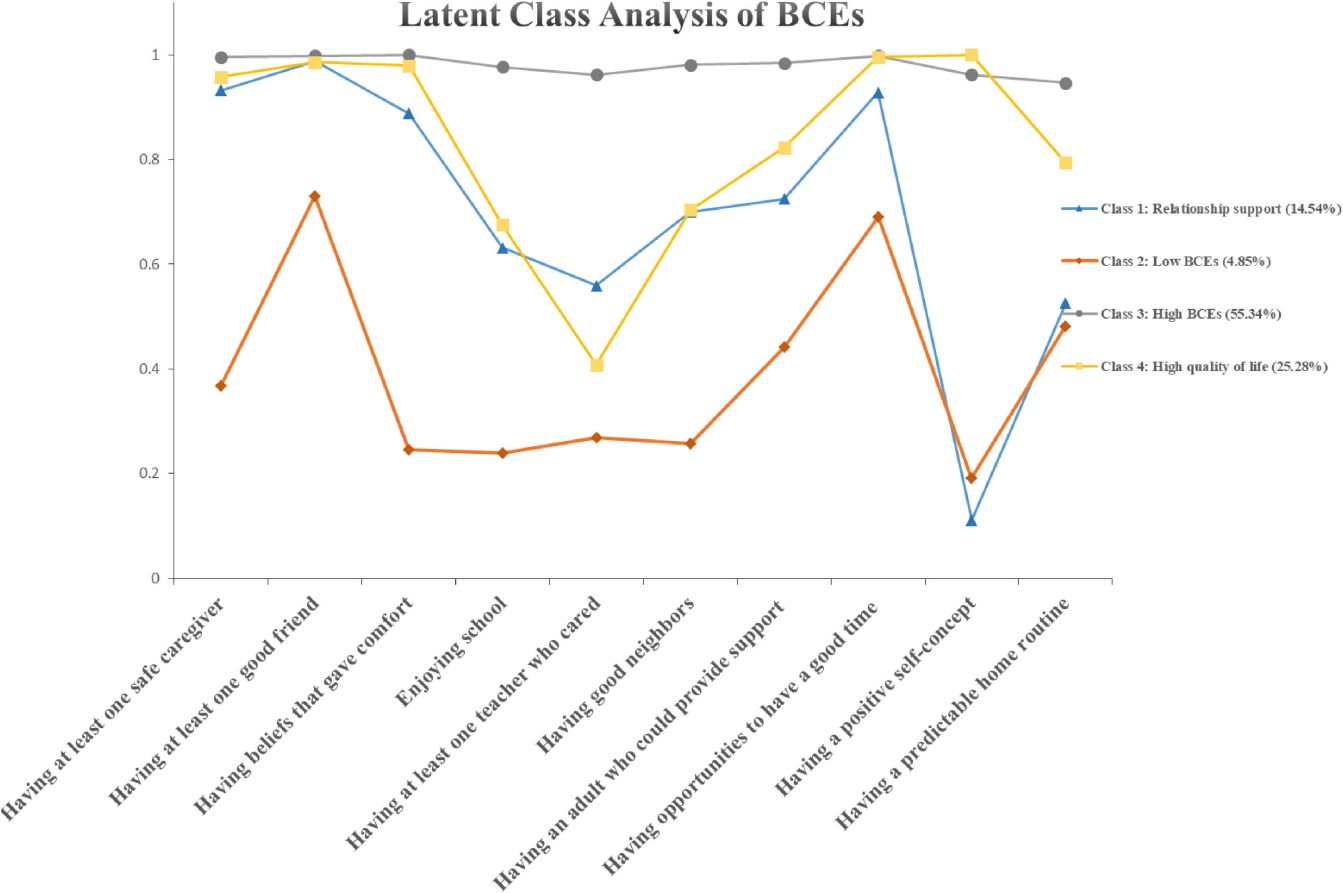
Probabilities of BCEs items by childhood experiences pattern derived by LCA Note: BCEs = benevolent childhood experience; LCA = latent class analysis

To clarify the specific classes in the LCA model, we would further classify them. According to Fig. 1, three distinctive classes were identified: class 1: emotional abuse (10.57%); class 2: high ACEs (0.55%); and class 3: low ACEs (88.88%). Those in the high ACEs category had a relatively high probability of ACEs exposure. Individuals in the emotional abuse class reported a considerably high possibility of endorsing exposure to emotional abuse (76.20%). Participants in the low ACEs class had low odds in all ACEs categories. According to Fig. 2, four distinctive classes were identified: class 1: relationship support (14.54%); class 2: low BCEs (4.85%); class 3: high BCEs (55.34%); and class 4: high quality of life (25.28%). Participants in the high BCEs Class were typical by high odds of all BCEs categories and in the high quality of life class reported considerably high likelihoods of exposures to feel comfortable with themselves (100%). Individuals in the relationship support class have at least one good friend.

### Associations between childhood experiences and depression among Chinese undergraduates

To explore the correlation between childhood experiences and depression among Chinese undergraduates, we conducted a multivariable logistic regression analysis. As illustrated in Table 3, model 1 demonstrated that bisexuality (OR = 1.53, 95%CI = 1.04–2.32) and other (OR = 1.90, 95%CI = 1.03–3.50) were positively related to depression. Based on model 1, model 2 added ACEs. Compared with the low ACEs class, the emotional abuse class’s OR was 3.82 (95%CI = 2.80–5.22) and was positively related to depression. Based on model 1, model 3 added BCEs. And model 3 indicated that the relationship support class (OR = 0.45, 95%CI = 0.24–0.84), high quality of life class (OR = 0.09, 95%CI = 0.05–0.16), and high BCEs class (OR = 0.05, 95%CI = 0.03–0.09) were notably negatively related to depression. Both ACEs and BCEs existed in model 4, and multivariable logistic regression consequences showed that emotional abuse class (OR = 2.39, 95%CI = 1.68–3.40) was the risk factor for depression. In contrast, the high quality of life class (OR = 0.11, 95%CI = 0.06–0.20) and high BCEs class (OR = 0.06, 95%CI = 0.04–0.12) were protective factors for depression.

**Table 3:** Associations between childhood experiences and depression among Chinese undergraduates

**Characteristics**	**Model 1**	**Model 2**	**Model 3**	**Model 4**
	**OR (95%CI)**	**OR (95%CI)**	**OR (95%CI)**	**OR (95%CI)**
**Gender**				
Male	1.11(0.90, 1.37)	1.13(0.90, 1.41)	1.17(0.92, 1.48)	1.18(0.92, 1.50)
Female	1	1	1	1
**Living expenses**				
<1000	1.26(0.62, 2.54)	1.12(0.54, 2.30)	1.05(0.49, 2.25)	0.98(0.4, 2.12)
1000–2000	1.14(0.64, 1.95)	1.05(0.59, 1.86)	0.90(0.49, 1.64)	0.89(0.48, 1.63)
2001–3000	1.27(0.69, 2.36)	1.28(0.65, 2.29)	1.05(0.51, 2.04)	1.06(0.54, 2.07)
>3000	1	1	1	1
**Only-child**				
Yes	1	1	1	1
No	1.17(0.95, 1.44)	1.19(0.97,1.48)	1.11(0.88, 1.39)	1.12(0.89, 1.42)
**Residence**				
Urban	1.08(0.88, 1.33)	1.07(0.86, 1.32)	1.10(0.87, 1.38)	1.09(0.86, 1.37)
Rural	1	1	1	1
**Sexual orientation**				
Heterosexuality	1	1	1	1
Homosexuality	1.53(0.81, 2.90)	1.50(0.78, 2.88)	1.38(0.68, 2.82)	1.38(0.67, 2.81)
Bisexuality	1.55(1.04, 2.32)*	1.39(0.92, 2.10)	1.32(0.84, 2.07)	1.22(0.78, 1.92)
Other	1.90(1.03, 3.50)*	1.79(0.96, 3.36)	1.86(0.94, 3.66)	1.83(0.92, 3.63)
**ACEs**				
High ACEs (0.55%)		0.61(0.13, 2.92)		0.86(0.18, 4.15)
Emotional abuse (10.57%)		3.82(2.80, 5.22)***		2.39(1.68, 3.40)***
Low ACEs (88.88%)		1		1
**BCEs**				
High BCEs (55.34%)			0.05(0.03, 0.09)***	0.06(0.04, 0.12)***
High quality of life (25.28%)			0.09(0.05, 0.16)***	0.11(0.06, 0.20)***
Relationship support (14.54%)			0.45(0.24, 0.84)*	0.53(0.28, 1.08)
Low BCEs (4.85%)			1	1

Note: ACEs = adverse childhood experience; BCEs = benevolent childhood experience; *P < 0.05, **P < 0.01, ***P < 0.001

### Associations between childhood experiences and suicidal ideation among Chinese undergraduates

As illustrated in Table 4, model 1 demonstrated that homosexuality (OR = 3.04, 95%CI = 1.36–6.81) and bisexuality (OR = 3.43, 95%CI = 2.10–5.59) were positively related to suicidal ideation. Based on model 1, model 2 added ACEs. Compared with the low ACEs class, the emotional abuse class’s OR was 5.77 (95%CI = 3.97–8.38) was positively related to suicidal ideation. Based on model 1, model 3 added BCEs. Model 3 indicated that the high quality of life class (OR = 0.22, 95%CI = 0.12–0.40) and the high BCEs class (OR = 0.15, 95%CI = 0.09–0.26) were negatively related to suicidal ideation. Both ACEs and BCEs existed in model 4, and multivariable logistic regression consequences showed that the emotional abuse class (OR = 4.07, 95%CI = 2.73–6.07) and high ACEs class (OR = 5.93, 95%CI = 1.19–29.66) were the risk factors of suicidal ideation. In contrast, the high quality of life class (OR = 0.34, 95%CI = 0.18–0.63) and high BCEs class (OR = 0.25, 95%CI = 0.36–0.45) were protective factors of suicidal ideation.

**Table 4:** Associations between childhood experiences and suicidal ideation among Chinese undergraduates

**Characteristics**	**Model 1**	**Model 2**	**Model 3**	**Model 4**
	**OR (95%CI)**	**OR (95%CI)**	**OR (95%CI)**	**OR (95%CI)**
**Gender**				
Male	1	1	1	1
Female	1.27(0.87, 1.85)	1.26(0.85, 1.86)	1.25(0.84, 1.84)	1.20(0.81, 1.79)
**Living expenses**				
<1000	2.62(0.78, 8.83)	2.39 (0.68, 8.48)	2.43(0.69, 8.59)	2.36(0.66, 8.50)
1000–2000	1.73(0.61, 4.94)	1.74(0.59, 5.16)	1.54(0.52, 4.58)	1.56(0.52, 4.66)
2001–3000	1.15(0.31, 3.68)	1.18(0.36, 3.91)	1.03(0.31, 3.42)	1.05(0.31, 3.51)
>3000	1	1	1	1
**Only-child**				
Yes	1	1	1	1
No	1.03(0.73, 1.45)	1.09(0.77, 1.55)	1.96(0.67, 1.36)	1.03(0.72, 1.47)
**Residence**				
Urban	1.36(0.96, 1.93)	1.34(0.93, 1.92)	1.41(0.98, 2.01)*	1.37(0.95, 1.98)
Rural	1	1	1	1
**Sexual orientation**				
Heterosexuality	1	1	1	1
Homosexuality	3.04(1.36, 6.81)**	3.08(1.32, 7.20)*	2.80(1.19, 6.57)*	2.96(1.24, 7.08)*
Bisexuality	3.43(2.10, 5.59)***	3.00(1.79, 5.03)***	3.16(1.89, 5.28)***	3.05(1.80, 5.16)***
Other	2.21(0.96, 5.09)	2.01(0.83, 4.85)	2.04(0.84, 4.95)	2.00(0.79, 5.06)
**ACEs**				
High ACEs (0.55%)		4.28(0.86, 21.37)		5.93(1.19, 29.66)*
Emotional abuse (10.55%)		5.77(3.97, 8.38)***		4.07(2.73, 6.07)***
Low ACEs (88.88%)		1		1
**BCEs**				
High BCEs (55.34%)			0.15(0.09, 0.26)***	0.25(0.36, 0.45)***
High quality of life (25.28%)			0.22(0.12, 0.40)***	0.34(0.18, 0.63)***
Relationship support (14.54%)			0.77(0.43, 1.35)	1.08(0.59, 1.96)
Low BCEs (4.85%)			1	1

Note: ACEs = adverse childhood experience; BCEs = benevolent childhood experience; *P < 0.05, **P < 0.01, ***P < 0.001

## Discussion

This study aims to investigate the co-occurrence pattern of ACE and BCEs exposure among Chinese undergraduates as well as the relationship between latent classes of ACEs and BCEs and Chinese undergraduates’ mental health. To the best of our knowledge, few studies used LCA to discover the unique pattern of BCEs and to explore the association between ACEs and BCEs and psychological distress simultaneously. Below we present the main findings and discuss limitations and future directions.

### Latent classes and the relationship between ACEs pattern and psychological distress

Our research found that ACEs patterns were classified into three latent classes: high ACEs, emotional abuse, and low ACEs. Furthermore, we discovered that adolescents exposed to high levels of ACEs were more likely to experience psychological symptoms than those exposed to lower levels of ACEs. A growing body of research has provided clear evidence that the empirical identification of discrete classes of adolescents who support similar ACEs patterns is feasible [36–38]. Although the existing studies used different ACEs indicators, which led to inconsistency of ACEs classes, they had found various ACEs classes, usually ranging from low ACEs to high ACEs classes [36–38]. Similarly, other LCA studies have found a class defined by experiences of emotional abuse [39, 40]. However, because each study contained different ACEs, it is not easy to compare these classes directly. It is worth noting that even classes with similar names in other studies may differ significantly. For example, the high ACEs class in our study was more likely to support household mental illness (100%) compared to the high ACEs class in the Merians et al. study (74–81%) [41]. Overall, adolescents with high ACEs had a worse psychological situation than those with low ACEs. Our results are generally consistent with previous studies.

According to literature records, ACEs are related to various psychological, physiological, behavioral, and functional problems [42]. At present, the results of population research show that besides psychological problems, ACEs may have a long-term impact on individuals’ social cognition [43]. ACEs are well-known risk factors for early-onset depression and chronic refractory depression [44–47]. This finding can be explained by stress sensitivity theory [48]. The theory suggests that ACEs can reduce the threshold of depression, which leads individuals to severe depression and depressive thoughts [49, 50]. Concerning suicidal ideation risk, a landmark ACEs study found a dose-response gradient between exposure to child maltreatment and neglect and lifetime suicidal ideation attempts [51, 52]. Previous studies using nationally representative data from the United States and Canada have confirmed that ACEs are closely related to the prevalence of suicidal ideation in these countries. For example, if you have not been exposed to ACEs, the suicidal ideation of the general population in Canada will be reduced by more than 50% [53–55]. Neglect of ACEs will lead to serious injuries in adolescence. The consequences of a child being constantly humiliated, insulted, belittled, rejected, or isolated can be severe. These signs indicate that the potential protective factors become crucial in ACEs.

In a word, ACEs deprive people of the proper environment to develop these skills, making them more vulnerable to risk factors that can lead to maladjustment. Therefore, it is necessary to prevent ACEs exposure or help build adaptive capacity to overcome the aspects of ACEs exposure. For those with ACEs, the interventions to improve parenting skills, strengthen parent-child relationships, and develop children’s resilience can help mitigate the harmful effects of ACEs.

### Latent classes and the relationship between BCEs pattern and psychological distress

According to our findings, BCEs patterns can be classified into four latent classes: high BCEs, positive quality of life, relationship support, and low BCEs. Approximately 55.34% of participants were categorized into the high BCEs. A study in America that investigated BCEs patterns discovered that the highest BCEs pattern was the largest class [56]. Class structure differed for adolescents, with generally lower proportions of adolescents in class 1 (relationship support) and class 4 (positive quality of life) experiencing being cared for by at least one teacher and a lower likelihood of positive self-concept in class 1 (relationship support) and class 2 (low BCEs). It may be because the teacher has to deal with dozens of students and has limited time and energy to take care of every student in the class. Contemporary adolescents commonly lack self-confidence due to their over-ambitious pursuits, resulting in a lower positive self-concept [57].

In the study of the relationship between BCEs and adolescent depression and suicidal ideation, multivariable logistic regression consequences showed that the high BCEs class is a protective factor of mental health. In addition, the more BCEs, the better the mental health. Even in adversity, BCEs can be used as a protective factor, consistent with existing studies [56, 58]. BCE can enhance the cognition and emotional processing of experience, promoting the reassessment of stress events in a psychologically adaptive way [59–61]. BCEs are a crucial factor in acquiring developmental competencies such as resilience, self-control, and emotional regulation, which are critical for adolescents to build healthy relationships.

In short, BCEs provide the necessary security for people to acquire developmental skills essential to positive adjustment. Therefore, it is required to advocate the development of BCEs.

### Limitation

This study includes limitations that are important to note. First, because of the cross-sectional nature, we can’t attribute the relationship between childhood experience and distress to causality. Second, we used retrospective measures of childhood experiences, which could be influenced by respondents’ memory and selectivity bias. Third, as the participants are primarily from three provinces in China and are composed of college students, the sample’s representativeness may be limited. Fourth, this research depends on the participants’ views of themselves. Future research may have feedback from more sources, such as family and friends, and should be conducted on a broader population to explore ACEs and BCEs.

## Conclusion

The current study investigated clusters of childhood experiences in undergraduate students. The classification of children based on their childhood experiences provides insight into the complex nature of early childhood experiences and may help guide the improvement of practices. Those in these clusters differed in terms of whether they experienced childhood adversity and benevolent experiences, but more importantly, in terms of the types of experiences they encountered. Our research results show that high ACEs and emotional abuse patterns were significantly associated with psychological distress. In contrast, more BCEs were associated with better mental health. These findings point out that it is more necessary to identify and support victims of ACEs, and it is urgent to increase BCEs in early childhood.

## Data Availability

The raw data supporting the conclusions of this article will be made available by the authors without undue reservation.
